# Cancer lung: An unusual presentation

**DOI:** 10.4103/0971-5851.65343

**Published:** 2009

**Authors:** Shankar L. Jakhar, Rohitashwa Dana, D. P. Punia

**Affiliations:** *Department of Radiotherapy, Mathura Das Mathur Hospital, Dr. Sampurnanand Medical College, Jodhpur, Rajasthan, India*

**Keywords:** *Bone metastasis*, *cancer lung*, *phalanx*

## Abstract

Phalanx bone metastasis as the initial manifestation of lung cancer is a rare presentation. A 70-year-old man presented with swelling and pain in his right ring finger. He had no other complaints or abnormal findings on clinical examination. A right hand radiograph showed an osteolytic lesion in the first phalanx of the ring finger. Fine needle aspiration cytology of the swelling suggested a metastatic adenocarcinoma. A skeletal survey, hematological, biochemical, and other radiological tests were found to be normal, except for an opacity seen in the right lung midzone. A bronchoscopic biopsy revealed adenocarcinoma of the lung.

## INTRODUCTION

Bone metastases from the lung cancer may occur early in the clinical course and are usually discovered to exist with pain.[1,2] The spine and ribs are often the earliest sites of bone metastases, whereas, the skull, femur, humerus, and scapula are involved later.[3] Metastases to the hands are rare events with around 200 cases reported in the literature.[4–8] They comprise only 0.1% of all osseous metastases. The terminal phalanges are the most frequent sites of metastasis, followed by the metacarpals and the proximal phalanges.[5,6] Reporting of phalange metastasis as the initial manifestation in lung cancer is an unusual presentation.

## CASE REPORT

**Figure 1:**
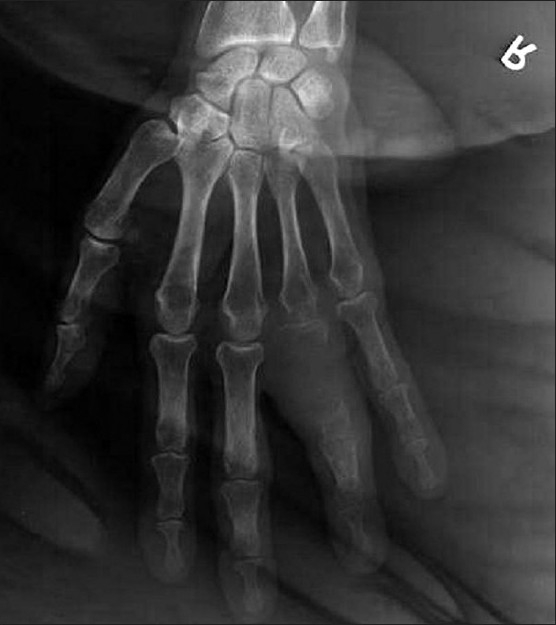
X-ray showing destruction of the first phalanx of the ring finger

**Figure 2:**
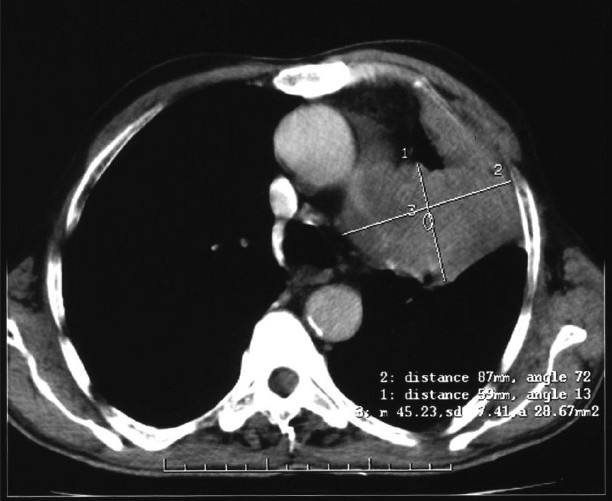
Chest CT scan showing left mid lobe mass

**Figure 3:**
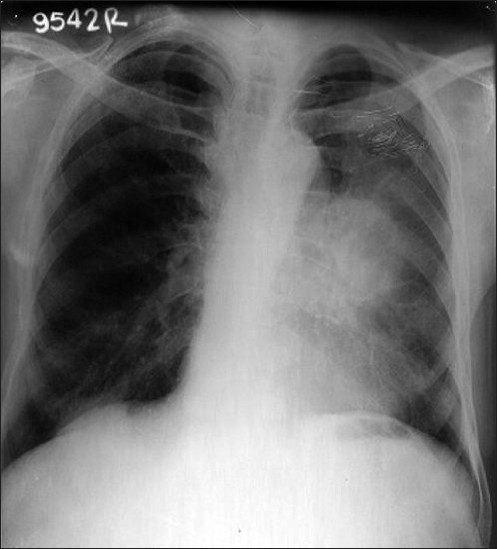
X-ray chest showing mass in left lung mid zone

A 70-year-old man presented with a complaint of swelling and pain in the right ring finger. The swelling gradually increased in size and became painful over a one-month period. He had no other complaints. Physical examination revealed no clinical findings other than swelling in the right ring finger proximally. The swelling was of mixed consistency, tender to palpation, and it restricted finger movement. A radiograph of the right hand showed an osteolytic lesion in the proximal phalanx of the ring finger [Figure 1]. FNAC of the swelling suggested metastatic adenocarcinoma. The hematological and biochemical profiles were normal. Ultrasonography of the whole abdomen and the skeletal survey was within normal limits [Figure 2]. A plain chest radiograph showed a mass lesion in the midzone of the right lung [Figure 3]. Bronchoscopic biopsy and aspiration cytology revealed adenocarcinoma of the lung. A bone scan showed increased uptake in the proximal phalanx of the right ring finger. We planned systemic chemotherapy and palliative radiotherapy to the finger with a nonsteroidal analgesic for relieving pain.

## DISCUSSION

Bone metastasis in distal parts of the extremities is very rare.[9–11] The first clinical description of peripheral bone metastases to the small bones of the hand was reported in a case of breast cancer, metastatic to the metacarpals, by Handley,[12] in 1960. The most common primary malignancy that metastasizes to the hand is lung carcinoma (42%) followed by the breast and kidney, each of which accounts for 11%.[5,12] The most common site of metastatic deposit to the hand is the distal phalanx. The incidence of metastasis in hand bones is 17% in metacarpals, 66% in phalanges, and 17% in carpal bones.[13] Bouvier *et al*. had described 256 cases of peripheral bone metastases in 1971, however, the series includes the bones of the forearm and legs. Peripheral bone metastases are mostly associated with disseminated disease, but sometimes account for the first symptom of the neoplasm. Patients may first receive medical attention as the result of skeletal metastasis from an unknown primary tumor. Imaging studies in such individuals may help to identify the primary lesion. A chest CT scan may occasionally be helpful in diagnosing lung cancer, which is not obvious on a plain chest radiograph. Therefore, a chest CT scan and bronchoscopy are undertaken when there is a clinical indication. In a reported case, at the time of initial diagnosis the right hand ring finger lesion was recognized as a primary bone tumor in a radiograph, because of the unusual localization of the bone metastasis. Among lung cancers, adenocarcinomas are more heterogeneous in progression than other cell types of lung cancer. In patients with advanced lung cancer, the major goal of treatment is recovery of the performance status of the patient and relief from pain. In a certain percent of cases, however, intensive systemic chemotherapy would be indicated as an adjuvant to local therapy such as radiotherapy and / or surgical procedures. Radiotherapy of 800 cGy single fraction at the metastatic bone site and oral analgesics improved the quality of life in our patient.

## CONCLUSIONS

When unusual bone metastases are found in the absence of a primary tumor, the investigation must include a chest radiograph, and effective management of these cases could improve the patient‘s quality of life.
